# Presumptive identification of *Candida *species other than *C. albicans*, *C. krusei*, and *C. tropicalis *with the chromogenic medium CHROMagar Candida

**DOI:** 10.1186/1476-0711-5-1

**Published:** 2006-01-03

**Authors:** Duane R Hospenthal, Miriam L Beckius, Karon L Floyd, Lynn L Horvath, Clinton K Murray

**Affiliations:** 1 Department of Medicine, Brooke Army Medical Center, Fort Sam Houston, Texas, USA; 2 Department of Clinical Investigation, Brooke Army Medical Center, Fort Sam Houston, Texas, USA

## Abstract

**Background:**

CHROMagar Candida (CaC) is increasingly being reported as a medium used to differentiate *Candida albicans *from non-*albicans Candida *(NAC) species. Rapid identification of NAC can assist the clinician in selecting appropriate antifungal therapy. CaC is a differential chromogenic medium designed to identify *C. albicans*, *C. krusei*, and *C. tropicalis *based on colony color and morphology. Some reports have proposed that CaC can also reliably identify *C. dubliniensis *and *C. glabrata*.

**Methods:**

We evaluated the usefulness of CaC in the identification of *C. dubliniensis*, *C. famata*, *C. firmetaria*, *C. glabrata*, *C. guilliermondii*, *C. inconspicua*, *C. kefyr*, *C. lipolytica*, *C. lusitaniae*, *C. norvegensis*, *C. parapsilosis*, and *C. rugosa*.

**Results:**

Most NAC produced colonies that were shades of pink, lavender, or ivory. Several isolates of *C. firmetaria *and all *C. inconspicua *produced colonies difficult to differentiate from *C. krusei*. Most *C. rugosa *isolates produced unique colonies with morphology like *C. krusei *except in a light blue-green color. *C. glabrata *isolates produced small dark violet colonies that could be differentiated from the pink and lavender colors produced by other species. All seventeen isolates of *C. dubliniensis *produced green colonies similar to those produced by *C. albicans*.

**Conclusion:**

*C. glabrata *and *C. rugosa *appear distinguishable from other species using CaC. Some NAC, including *C. firmetaria *and *C. inconspicua*, could be confused with *C. krusei *using this medium.

## Background

*Candida albicans *once accounted for most serious nosocomial candidal infections. Over the last decade *Candida *infections, specifically candidemia, due to *C. albicans *have declined [1]. Other non-*albicans Candida *species (NAC) are now responsible for about half of candidemias and other deep *Candida *infections [2-4]. Most NAC infections are caused by *C. glabrata*, *C. parapsilosis*, or *C. tropicalis*, although in total over a dozen NAC have been reported to cause candidemia and other invasive infections [5-8]. In addition to deoxycholate and lipid formulations of amphotericin B, clinicians now must chose from azoles with differing spectrums of activity (fluconazole, itraconazole, and voriconazole) and echinocandin antifungal agents. *In vitro *testing has revealed that there are clear differences among the various NAC in their susceptibility to specific drugs. Rapid, reliable identification to species is now needed more than ever for clinicians to make treatment choices.

Identification of yeast pathogens by traditional methods requires several days and specific mycological media. Chromogenic media contain chromogenic substrates which react with enzymes secreted by the target microorganisms to yield colonies of varying colors. CHROMagar Candida (CaC) is one such medium that, per its manufacturer (CHROMagar Microbiology, Paris, France, ), can identify three candidal yeasts, *C. albicans *(green colonies), *C. tropicalis *(steel blue colonies), and *C. krusei *(fuzzy, rose colored colonies) after 48 hours of incubation at 30–37°C. Independent groups have reported success with CaC in differentiating *C. dubliniensis *from *C. albicans *[9,10]. Investigators have also reported the ability to distinguish *C. glabrata *from other species [11-14], although this has been contested by other authors [15-17].

We studied the appearance of 180 isolates of 12 *Candida *species on CaC to determine whether these more rare species could be reproducibly distinguished from each other with this medium.

## Methods

Clinical yeast isolates were employed for all testing, predominately from our facility. Additional isolates were provided by the Fungus Testing Laboratory, University of Texas Health Science Center at San Antonio, Texas. Yeasts tested included *C. dubliniensis *(17 isolates), *C. famata *(11 isolates), *C. firmetaria *(12 isolates), *C. glabrata *(38 isolates), *C. guilliermondii *(10 isolates), *C. inconspicua *(6 isolates), *C. kefyr *(9 isolates), *C. lipolytica *(10 isolates), *C. lusitaniae *(15 isolates), *C. norvegensis *(3 isolates), *C. parapsilosis *(34 isolates), and *C. rugosa *(15 isolates). Five isolates each of *C. albicans*, *C. krusei*, and *C. tropicalis *were used as controls. All isolates were identified by standard clinical practices as previously described [18].

Inocula from clinical isolates stored at -70°C were transferred into yeast extract peptone (YEP) broth (Bacto Peptone, BD, Sparks, MD) and incubated at 30°C in a rocking incubator for up to 96 hours. Recovered yeasts were then streaked for isolation onto Sabouraud dextrose agar (SDA) plates (BD, Sparks, MD) and incubated at 30°C for 24–48 hours and subcultured a second time prior to inoculation in duplicate onto commercially prepared CHROMagar Candida (CaC) plates (BD, Sparks, MD). Inoculated CaC plates were incubated in parallel at 30°C and 37°C. Two independent readers, blinded to species inoculated, observed each set of plates for color and colony morphology daily for 7 days. Following the initial blinded study, select isolates were repeated in an unblinded fashion in parallel with the control isolates for direct comparison.

## Results

Individual isolates produced the same colors at both incubation temperatures. In general, these colors appeared more intense at 37°C and intensified daily at both temperatures, peaking at 72 hours. Most of the NAC (*C. famata*, *C. firmetaria*, *C. guilliermondii*, *C. inconspicua*, *C. kefyr*, *C. lipolytica*, *C. lusitaniae*, *C. norvegensis*, and *C. parapsilosis*) tested produced variable shades of ivory, pink, and lavender (Table 1). *C. parapsilosis *isolates were the most varied of all species tested, in both color and colony morphology. *C. kefyr *commonly produced large colonies with centers that deepened to a brownish color with duration of incubation. *C. firmetaria *and *C. inconspicua *isolates produced large flat colonies. Most of the *C. firmetaria *and all of the *C. inconspicua *were pink with pale borders and rough in texture, indistinguishable from the color and morphology of *C. krusei*. *C. rugosa *most commonly (eleven of 15 isolates) produced light blue-green colonies with pale borders, similar in morphology (large, flat) to *C. krusei*. The minority of isolates grew as small to medium pink colonies not distinguishable from many other species.

**Table 1:** The appearance of *Candida *species grown on CHROMagar Candida.

	Colony characteristics		
Species	Isolates (no.)	Color	Morphology
*C. dubliniensis*	17	Green	--^a^
*C. famata*	11	Pink to lavender	--
*C. firmetaria*	12	Pink to lavender, pale border	Some flat, rough; others waxy^b^
*C. glabrata*	38	Dark violet^c^	Small to medium, smooth, convex, creamy
*C. guilliermondii*	10	Pink to lavender	--
*C. inconspicua*	6	Pink to lavender, pale borders	Flat, rough^d^
*C. kefyr*	9	Pink to lavender, often with darkened centers	Large, rough
*C. lipolytica*	10	Ivory to pink	Large, flat, rough, wrinkled^b^
*C. lusitaniae*	15	Pink to lavender	Some waxy
*C. norvegensis*	3	Ivory to pink	Large, rough^b^
*C. parapsilosis*	34	Ivory to pink to lavender	Small to medium, smooth to wrinkled
*C. rugosa*	15	Light blue-green, pale border^e^	Medium to large, flat

*C. albicans*	5	Green	--
*C. krusei*	5	Pink, pale borders	Medium to large, flat, rough
*C. tropicalis*	5	Steel blue, purple diffusion	--

^a^morphology if not noted – medium size, smooth, convex, creamy

^b^some isolates produce colonies indistinguishable from *C. krusei*

^c^dark pink colonies at 24 hours, darkening with time; colonies commonly had thin pale borders and produced pigment which diffused into medium

^d^all isolates indistinguishable from *C. krusei*

^e^Typically produced light blue-green strains with morphology similar to *C. krusei*, but some (4) produced small to medium smooth, convex, creamy pink colonies.

*C. glabrata *was readily identifiable using CaC, producing small, convex, dark pink to violet colonies. Peak color intensity was noted at 72 hours when incubated at 30°C and at 48 hours when incubated at 37°C. *C. glabrata *isolates typically produced colonies with thin pale borders and a deep violet pigment that diffused into the medium. Unfortunately, neither of these features were universal. Our readers could not distinguish the medium to dark green colonies produced by *C. dubliniensis *from those of *C. albicans *at either temperature or any duration, except when directly compared to each other on repeat testing.

## Discussion

Amphotericin B, fluconazole, and caspofungin are currently the three antifungal agents most commonly used to treat candidemia and invasive candidiasis. This practice is supported by FDA approval and the most recent Infectious Diseases Society of America guidelines [19]. While all these agents have been used effectively in clinical studies, these studies included mostly patients with *C. albicans *infections; thus their efficacy against NAC is not necessarily universal or well known. *In vitro *study has shown decreased susceptibility to amphotericin B in isolates of *C. famata*, *C. guilliermondii*, *C. inconspicua*, *C. kefyr*, *C. krusei*, *C. lusitaniae*, and *C. rugosa *and to fluconazole in isolates of *C. famata*, *C. firmetaria*, *C. guilliermondii*, *C. inconspicua*, *C. krusei*, and *C. lusitaniae *[7,8]. The MICs of caspofungin have been demonstrated to be higher in *C. famata*, *C. guilliermondii*, and *C. parapsilosis *isolates [20]. The number of antifungal agents available continues to increase in the setting of a shift of candidal infections to those caused by non-*albicans *species. Because of this, identification to species and increased use of susceptibility testing has become necessary to appropriately select which agent to use [21].

CHROMagar Candida is a chromogenic medium that is advertised as able to identify *C. albicans*, *C. krusei*, and *C. tropicalis*. With the increasing incidence of human disease being produced by the less common *Candida *species, we were interested in testing the performance of this medium with the less common agents of candidiasis. We found, as previously reported, that most of these rarer *Candida *species and *C. parapsilosis *produced typical convex, creamy yeast colonies in shades of pink, lavender, and less commonly ivory, not distinguishable from each other (and often not consistent between isolates of the same species). *C. parapsilosis*, one of the most commonly recovered NAC, produced the widest range of colors and morphologies, making it impossible to identify using this medium.

Many of the rare NAC produced morphology and colors similar to those seen with *C. krusei *on this medium. We found individual isolates of *C. lipolytica *and *C. norvegensis*, many isolates of *C. firmetaria*, and all tested *C. inconspicua *were indistinguishable from *C. krusei*. Some that were distinguishable, produced similar color as that of *C. krusei*, with the pale border, but with waxy colonies. Most of these species share reduced susceptibility to fluconazole that is common with *C. krusei*.

We confirmed our previous observation that *C. rugosa *appears to typically produce a readily identifiable and unique color/colony type on CaC [12,22]. Eleven of 15 isolates of this organism produced the same light blue-green color and a colonial morphology similar to that of *C. krusei*. *C. rugosa *has been shown in clinical reports and by *in vitro *testing to be less susceptible to amphotericin B [7,8,23]. Rapid identification of this NAC is of great importance to allow provision of appropriate therapy to patients.

As previously reported by our group and others, this study did find *C. glabrata *to be readily distinguishable from other species that produced pink to lavender colonies on CaC. With the use of larger numbers of isolates, we again noted that this species produced smaller colonies starting out as dark pink hues, becoming dark violet with time, commonly with a small diffusion of dark violet pigment into the surrounding agar and a thin pale border [12,22]. This was most apparent after prolonged incubation (72 hours) at the lower temperature suggested by the manufacturer (30°C), or after 48 hours when incubated at 37°C.

Our readers could not distinguish *C. dubliniensis *from *C. albicans *when observed in a blinded fashion. The isolates we tested were somewhat darker, especially at the higher incubation temperature, but this was only obvious when CaC culture was performed in parallel to *C. albicans *controls. The report by Kirkpatrick et al. [9] that made the initial observation that *C. dubliniensis *produce darker green colonies with CaC did this with primary isolation of clinical materials onto the medium. Our isolates were not from primary samples and thus may have been affected by storage conditions and repeated subculturing. Odds and Davidson reported that they could differentiate *C. dubliniensis *from *C. albicans *using stored isolates, but this was best shown after 72 hours of incubation at 37°C [10].

## Conclusion

In our hands CHROMagar Candida shows good potential as a screening medium to rapidly identify potentially azole-resistant as well as amphotericin B-resistant species, including *C. krusei*, *C. glabrata*. *C. rugosa*, and *C. inconspicua*. Identification of *C. dubliniensis *appears to have some limitations based on our study, but CaC appears (from other studies) to also hold this potential when used as a primary medium. We would suggest inclusion of a *C. albicans *control plate if this medium is used as a primary fungal recovery medium to help increase the presumptive identification of *C. dubliniensis*. Combined with confirmation of speciation by standard methods and knowledge of the local antifungal susceptibility patterns of these species, CaC may be a useful adjunctive medium for use in the clinical laboratory.

## Competing interests

The author(s) declare that they have no competing interests.

## Authors' contributions

DH and LL had primary responsibility for study design. MB maintained isolates and prepared plates for observation. DH and KF served as readers for the blinded plate readings. All authors assisted in the unblinded reading of the plates and preparation of the manuscript. All authors have read and approved the final manuscript.
